# Supplementary material for: Evidence for a dual-process account of over-imitation: Children imitate anti- and prosocial models equally, but prefer prosocial models once they become aware of multiple solutions to a task
Source: PLoS One. 2021 Sep 16;16(9):e0256614. doi: 10.1371/journal.pone.0256614 (PMC8445421; doi:10.1371/journal.pone.0256614)
Supplement: S1 Text — (DOCX) [file pone.0256614.s001.docx]

**Supplementary material for:**

**“Evidence for a dual-process account of over-imitation: Children imitate anti- and prosocial models equally, but prefer prosocial models once they become aware of multiple solutions to a task”**

**S1 Text. Detailed description statistical analysis and results**

We applied one main logistic Generalized Linear Mixed Model (GLMMs; [1]) fitted via maximum likelihood using the statistical program R (version 3.4.3; [2]) together with the function ‘glmer’ of the package ‘lme4’ [3]. In the main GLMM we focused on the effect of the manipulations in the experimental conditions. Furthermore, we conducted four Generalized Liner Models (GLMs) for baseline comparisons using the function ‘glm’ of the same package. In the baseline comparisons we compared children’s tendency to over-imitate in the experimental conditions to children’s tendency to perform irrelevant actions in the baseline condition.

**Main GLMM.** We coded over-imitation, our response term, in a binary wat (0 = children did not perform the irrelevant action, 1 = children performed the irrelevant action). Thus, we set the error structure family to *binomial*, using a logit link function. Regarding the predictors, we were mainly interested in whether the interaction between condition and phase had a significant effect on children’s tendency to over-imitate. Since previous research has shown that not all types of actions are over-imitated to the same extend, we further wanted to see whether actions that involve physical contact with the puzzle box are less effected by children’s social motivations to imitate than actions that do not involve any physical contact with the box. Therefore, we included the three-way-interaction between condition, phase and action type into our main model. Prior research also revealed gender effects showing that boys over-imitate more than girls [4,5]. This is why we also checked whether children’s gender predicted their tendency to over-imitate. To account for repeated measurements, we also included the random effect for individual’s identity and the random slopes for phase and action type within individual identity in the model equation of the GLMM. To make results unconditional of the reference category of factor variables, we manually dummy coded and centered the factors phase and action types within the random slopes. The correlations of the random effects in the model were unidentifiable, so we excluded them from the model. This is indicated by the double pipe symbol (||). All statistical models were fitted with bound optimization by quadratic approximation (BOBYQA optimization, [6]). Taken together, this led to the following full model equation:

over-imitation _(yes/no)_ ~ (condition _(prosocial-antisocial/antisocial-prosocial)_ *

phase _(first-inefficient demo/second-efficient demo)_ *

action type _(contact/noncontact)_) +

child’s gender _(male/female)_ +

(1 + phase _manually_ _dummy coded and centered_ +

action type _manually_ _dummy coded and centered_ ||

individual identity)

In the next step, we checked whether assuming the absence of collinearity was justified. Generalized variance inflation factors (vif) were derived using the function *vif* of the R package *car* [7] applied to a standard linear model (using the function ‘lm’) excluding the interactions and random effects. This kind of analysis revealed that collinearity was no issue (largest vif = 1). We further checked the distribution of random effects visually, which fulfilled the assumption to be normally distributed. To estimate the stability of the model, we excluded the levels of random effects (individual identity) one at a time and compared the resulting estimates with those obtained from the model based on all data. All estimates can be seen as being stable (see S1 Table)

**S1 Table. Model stability test for full GLMM**

|  | original estimate | minimum estimate^1^ | maximum estimate^1^ |
| --- | --- | --- | --- |
| (Intercept) | -1.083 | -1.301 | -0.912 |
| condition_(antisocial-prosocial)_ ^(2)^ | -1.523 | -1.693 | -1.324 |
| phase _(phase 2)_ ^(2)^ | -1.501 | -1.739 | -1.27 |
| action type _(pseudo-instrumental)_ ^(2)^ | 2.129 | 1.928 | 2.444 |
| gender _(male)_ ^(2)^ | 0.525 | 0.407 | 0.676 |
| condition_(antisocial-prosocial)_: phase _(phase 2)_ ^(2)^ | 0.44 | 0.242 | 0.696 |
| condition_(antisocial-prosocial)_: action type _(pseudo-instrumental)_ ^(2)^ | 0.973 | 0.737 | 1.157 |
| phase _(phase 2)_: action type _(pseudo-instrumental)_ ^(2)^ | -0.29 | -0.636 | 0.058 |
| condition_(antisocial-prosocial)_: phase _(phase 2)_:  action type _(pseudo-instrumental)_ ^(2)^ | -0.134 | -0.455 | 0.202 |
| random intercept id | 1.675 | 1.547 | 1.764 |
| random slope phase within id | 0.551 | 0 | 0.709 |
| random slope action type within id | 1.269 | 0.996 | 1.372 |

*Note.*

^(1)^ We excluded the data of one participant at a time and ran the models again. The maximum and minimum estimates of all these models are shown for all fixed and random effects. All estimates can be considered to be stable.

^(2)^ Factors were dummy coded and had the following reference levels: condition: prosocial-antisocial, phase: phase 1, action type: disconnected action, gender: female; the estimates for the single predictors indicate the change from the response when the predictor changes from the references level to the in parentheses indicated level of the predictor.

To avoid an increased type 1 error risk due to multiple testing, we first tested the overall effect of the test predictors. Therefore, we compared the full model’s deviance with that of a null model comprising only the random intercept and the random slopes to examine whether the inclusion of the fixed effects provided a better fit to the data. The full model provided a significantly better fit to the data than the null model (*χ*^2^ = 77.984, *df* = 8, *p* < .0001, R^2^-like effect size: 0.63). The R^2^-like effect size was calculated with the function ‘r.squaredGLMM’ of the package ‘MuMIn’.

To determine the effects of each predictor we further compared the full model with the corresponding reduced models lacking only the predictor of interest. Thus, first we compared the full model with a model that was lacking the three-way-interaction of condition:phase:action type. This revealed that this interaction was not significant (*χ*^2^ = 0.015, *df* = 1, *p* = .901, Nagelkerke's R^2^ < .001). The effect sizes were calculated with the function ‘r.squaredLR’ of the package ‘MuMIn’. To test the effects of all possible two-way interactions, we fitted a new model that contained all two-way interactions (but not the three-way-interaction) and compared this model with three respective reduced models that lacked the interaction of interest. This revealed that none of the possible two way interactions was significant (condition:phase: χ2 = 0.426, df = 1, p = .514, Nagelkerke's R^2^ = .001, condition:action type: χ2 = 1.942, df = 1, p = .163, Nagelkerke's R^2^ = .006, phase:action type: χ2 = 0.430, df = 1, p = .512, Nagelkerke's R^2^ = .001). To extract the influence of the main effects, we fitted a model with only the main effects as fixed effects and compared this model with the respective reduced model in which we removed one fixed effect at the time. This revealed that the main effects phase (χ2 = 25.686, df = 1, p < .001, Nagelkerke's R^2^ = .07) and action type (χ2 = 44.167, df = 1, p < .001, Nagelkerke's R^2^ = .13) had a significant effect on children’s probability to over-imitate. Condition had no effect on children’s probability to over-imitate (*χ*^2^ = 2.483, *df* = 1, *p* = .115, Nagelkerke's R^2^ = .007). Also, gender had no effect on children’s over-imitation (*χ*^2^ = 1.047, *df* = 1, *p* = .306, Nagelkerke's R^2^ = .003). The results of these comparisons as well as the model estimates are shown in S2 Table.

**S2 Table. Estimates, standard errors (SE), confidence intervals, and likelihood ratio test output for the single effects** **for the full GLMM**

|  | estimates | SE | CI^(2)^_Lower_ | CI^(2)^_Upper_ | *χ*^2^ | *df* | *p* |
| --- | --- | --- | --- | --- | --- | --- | --- |
| (Intercept) | -1.083 | 0.568 | -2.278 | 0.009 |  |  |  |
| condition_(antisocial-prosocial)_ ^(2)^ | -1.523 | 0.722 | -3.032 | -0.139 | 2.483 | 1 | .115 |
| phase _(phase 2)_ ^(2)^ | -1.501 | 0.571 | -2.711 | -0.427 | 25.686 | 1 | <.001* |
| action type _(pseudo-instrumental)_ ^(2)^ | 2.129 | 0.602 | 1.008 | 3.418 | 44.167 | 1 | <.001* |
| gender _(male)_ ^(2)^ | 0.525 | 0.515 | -0.497 | 1.602 | 1.047 | 1 | .306 |
| condition_(antisocial-prosocial)_:  phase _(phase 2)_ ^(2)^ | 0.440 | 0.837 | -1.242 | 2.103 | 0.426 | 1 | .514 |
| condition_(antisocial-prosocial)_:  action type _(pseudo-instrumental)_ ^(2)^ | 0.973 | 0.811 | -0.617 | 2.626 | 1.942 | 1 | .163 |
| phase _(phase 2)_:  action type _(pseudo-instrumental)_ ^(2)^ | -0.290 | 0.750 | -1.774 | 1.191 | 0.430 | 1 | .512 |
| condition_(antisocial-prosocial)_: phase _(phase 2)_:  action type _(pseudo-instrumental)_ ^(2)^ | -0.134 | 1.073 | -2.242 | 1.998 | 0.015 | 1 | .901 |

*Note.*

^(1)^ Factors were dummy coded and had the following reference levels: condition: prosocial-antisocial, phase: phase 1, action type: disconnected action, gender: female; the estimates for the single predictors indicate the change from the response when the predictor changes from the references level to the in parentheses indicated level of the predictor.

^(2)^ Confidence intervals were derived using 1000 parametric bootstraps.

**p*<.05

Even though we did not find an interaction effect between condition and phase, we performed 4 post-hoc pairwise comparisons with the package ‘emmeans’. P-values were corrected with a multivariate adjustment (“mvt”). The results of these comparisons are reported in Table S3.

**S3 Table. Post-hoc pairwise comparisons**

|  | estimates | SE | CI^(2)^_Lower_ | CI^(2)^_Upper_ | *z-ratio* | *p_mvt-corrected_* |
| --- | --- | --- | --- | --- | --- | --- |
| prosocial-antisocial/phase 1 –  antisocial-prosocial/phase 1 | 1.036 | 0.578 | -0.389 | 2.46 | 1.792 | .233 |
| prosocial-antisocial/phase 2 –  antisocial-prosocial/phase 2 | 0.674 | 0.603 | -0.813 | 2.16 | 1.118 | .633 |
| prosocial-antisocial/phase 1 –  prosocial-antisocial/phase 2 | 1.644 | 0.416 | 0.620 | 2.67 | 3.955 | <.001* |
| antisocial-prosocial/phase 1 –  antisocial-prosocial/phase 2 | 1.283 | 0.405 | 0.284 | 2.28 | 3.166 | .006* |

For the comparisons with the baseline we created four subsets of our dataset. Each subset included the data of one trial of one condition and the baseline condition. We then performed 4 GLMs with the function ‘glm’ of the package ‘lme4’. Each GLM included only the predictor *condition.* Since this was a between-subject comparison no random effect structure was necessary. We checked the stability of all four models using the DFBETA values (see S4 Table).

**S4 Table. Model stability test for the models of the baseline comparisons**

|  | original estimate | minimum estimate^1^ | maximum estimate^1^ |
| --- | --- | --- | --- |
| **Phase 1 of the prosocial-antisocial condition and baseline** |  |  |  |
| (Intercept) | -1.721 | -1.735 | -1.672 |
| condition_(antisocial-prosocial)_ ^(1)^ | 1.864 | 1.815 | 1.884 |
| **Phase 2 of the prosocial-antisocial condition and baseline** |  |  |  |
| (Intercept) | -1.721 | -1.735 | -1.672 |
| condition_(antisocial-prosocial)_ ^(1)^ | 0.890 | 0.842 | 0.921 |
| **Phase 1 of the antisocial-prosocial condition and baseline** |  |  |  |
| (Intercept) | -1.721 | -1.735 | -1.672 |
| condition_(antisocial-prosocial)_ ^(1)^ | 1.322 | 1.273 | 1.343 |
| **Phase 2 of the antisocial-prosocial condition and baseline** |  |  |  |
| (Intercept) | -1.721 | -1.735 | -1.672 |
| condition_(antisocial-prosocial)_ ^(1)^ | 0.581 | 0.532 | 0.611 |

*Note.*

^(1)^ The factor condition was dummy coded and had the baseline as a reference level; the estimate for this predictor indicates the change from the response when the predictor changes from the references level to the in parentheses indicated level of the predictor.

To determine the effects of the predictor condition for each of those four models we compared each full model with the corresponding reduced models with the intercept only using a likelihood ratio test. First, we compared Phase 1 of the prosocial-antisocial condition with the baseline condition. These two conditions did differ significantly in regard to children’s probability to perform irrelevant actions (*χ*^2^ = 38.21, *df* = 1, *p* <.001, Nagelkerke's R^2^ = .216). Second, we compared Phase 2 of the prosocial-antisocial condition with the baseline condition. Here we also found a significant effect of condition (*χ*^2^ = 7.45, *df* = 1, *p* = .006, Nagelkerke's R^2^ = .050). Third, we compared Phase 1 of the antisocial-prosocial condition with the baseline condition and found again a significant difference (*χ*^2^ = 19.26, *df* = 1, *p* <.001, Nagelkerke's R^2^ = .108). Last, we compared Phase 2 of the antisocial-prosocial condition with the baseline condition, which turned out not to be significant (*χ*^2^ = 3.15, *df* = 1, *p* <.076, Nagelkerke's R^2^ = .020). Please see S5 Table.

**S5 Table. Estimates, standard errors (SE), confidence intervals, and likelihood ratio test output for the single effects for the GLMs for baseline comparisons**

|  | estimates | SE | CI^(2)^_Lower_ | CI^(2)^_Upper_ | *χ*^2^ | *df* | *p* |
| --- | --- | --- | --- | --- | --- | --- | --- |
| **Phase 1 of the prosocial-antisocial condition and baseline** |  |  |  |  |  |  |  |
| (Intercept) | -1.721 | 0.263 | -2.271 | -1.232 |  |  |  |
| condition_(antisocial-prosocial)_ ^(1)^ | 1.864 | 0.324 | 1.246 | 2.523 | 38.213 | 1 | <.001* |
| **Phase 2 of the prosocial-antisocial condition and baseline** |  |  |  |  |  |  |  |
| (Intercept) | -1.721 | 0.263 | -2.271 | -1.232 |  |  |  |
| condition_(antisocial-prosocial)_ ^(1)^ | 0.890 | 0.334 | 0.248 | 1.564 | 7.450 | 1 | .006* |
| **Phase 1 of the antisocial-prosocial condition and baseline** |  |  |  |  |  |  |  |
| (Intercept) | -1.721 | 0.263 | -2.271 | -1.232 |  |  |  |
| condition_(antisocial-prosocial)_ ^(1)^ | 1.322 | 0.318 | 0.716 | 1.967 | 19.264 | 1 | <.001* |
| **Phase 2 of the antisocial-prosocial condition and baseline** |  |  |  |  |  |  |  |
| (Intercept) | -1.721 | 0.263 | -2.271 | -1.232 |  |  |  |
| condition_(antisocial-prosocial)_ ^(1)^ | 0.581 | 0.333 | -0.059 | 1.251 | 3.153 | 1 | .076 |

*Note.*

^(1)^ Factors were dummy coded and had the following reference levels: condition: prosocial-antisocial, phase: phase 1, action type: disconnected action, gender: female; the estimates for the single predictors indicate the change from the response when the predictor changes from the references level to the in parentheses indicated level of the predictor.

^(2)^ Confidence intervals were derived using 1000 parametric bootstraps.

**p*<.0125 (*p*-value is Bonferroni corrected for four tests).

**References**

[1] Baayen RH, Davidson DJ, Bates DM. Mixed-effects modeling with crossed random effects for subjects and items. Journal of Memory and Language 2008;59:390–412. https://doi.org/10.1016/j.jml.2007.12.005.

[2] Team RC. R: A language and environment for statistical computing. Vienna, Austria: R Foundation for Statistical Computing; 2020.

[3] Bates D, Mächler M, Bolker B, Walker SC. Fitting linear mixed-effects models using lme4. ArxivOrg 2014. https://doi.org/10.1063/pt.5.028493.

[4] Frick A, Clément F, Gruber T. Evidence for a sex effect during overimitation: boys copy irrelevant modelled actions more than girls across cultures. Royal Society Open Science 2017;4:170367–13. https://doi.org/10.1098/rsos.170367.

[5] Schleihauf H, Pauen S, Hoehl S. Minimal group formation influences on over-imitation. Cognitive Development 2019;50:222–36. https://doi.org/10.1016/j.cogdev.2019.04.004.

[6] Powell MJD. The BOBYQA algorithm for bound constrained optimization without derivatives. ExercicescorrigesCom 2009.

[7] Fox J, Weisberg S. Multivariate linear models in r. an appendix to an r companion to applied regression. 2011.
